# Multimodal deep learning for COVID-19 prognosis prediction in the emergency department: a bi-centric study

**DOI:** 10.1038/s41598-023-37512-3

**Published:** 2023-07-05

**Authors:** Franca Dipaola, Mauro Gatti, Alessandro Giaj Levra, Roberto Menè, Dana Shiffer, Roberto Faccincani, Zainab Raouf, Antonio Secchi, Patrizia Rovere Querini, Antonio Voza, Salvatore Badalamenti, Monica Solbiati, Giorgio Costantino, Victor Savevski, Raffaello Furlan

**Affiliations:** 1 Internal Medicine, Humanitas Clinical and Research Center, IRCCS, Humanitas Research Hospital, Humanitas University, Via A. Manzoni, 56, 20089 Rozzano, Milan, Italy; 2 IBM, Milan, Italy; 3 Department of Biomedical Sciences, Humanitas University, Via Rita Levi Montalcini 4, Pieve Emanuele, Italy; 4 IRCCS Humanitas Research Hospital, Via A. Manzoni, 56, 20089 Rozzano, Milan, Italy; 5 Department of Medicine and Surgery, University of Milano-Bicocca, Milan, Italy; 6 Heart Rhythm Department, Clinique Pasteur, Toulouse, France; 7 Emergency Department, Humanitas Mater Domini, Castellanza, Varese, Italy; 8 IRCCS-Ospedale San Raffaele, Università Vita-Salute San Raffaele, Milan, Italy; 9 Emergency Department, IRCCS - Humanitas Clinical and Research Center, Via Manzoni 56, Rozzano, Italy; 10 Emergency Department, Fondazione IRCCS Ca’ Granda, Ospedale Maggiore, Milan, Italy; 11 AI Center, IRCCS - Humanitas Research Hospital, Via Manzoni 56, Rozzano, Italy

**Keywords:** SARS-CoV-2, Prognosis, Machine learning

## Abstract

Predicting clinical deterioration in COVID-19 patients remains a challenging task in the Emergency Department (ED). To address this aim, we developed an artificial neural network using textual (e.g. patient history) and tabular (e.g. laboratory values) data from ED electronic medical reports. The predicted outcomes were 30-day mortality and ICU admission. We included consecutive patients from Humanitas Research Hospital and San Raffaele Hospital in the Milan area between February 20 and May 5, 2020. We included 1296 COVID-19 patients. Textual predictors consisted of patient history, physical exam, and radiological reports. Tabular predictors included age, creatinine, C-reactive protein, hemoglobin, and platelet count. TensorFlow tabular-textual model performance indices were compared to those of models implementing only tabular data. For 30-day mortality, the combined model yielded slightly better performances than the tabular fastai and XGBoost models, with AUC 0.87 ± 0.02, F1 score 0.62 ± 0.10 and an MCC 0.52 ± 0.04 (*p* < 0.32). As for ICU admission, the combined model MCC was superior (*p* < 0.024) to the tabular models. Our results suggest that a combined textual and tabular model can effectively predict COVID-19 prognosis which may assist ED physicians in their decision-making process.

## Introduction

According to data from the World Health Organization (WHO), the new severe acute respiratory syndrome coronavirus 2 (SARS-CoV-2)^1^ has infected over 532 million individuals globally and caused over 6 million deaths^1^.

The rapid spread of the virus necessitated an immediate adaptation of the health care system as intensive care units (ICUs) quickly reached capacity^2^. Due to the serious complications associated with Coronavirus Disease 2019 (COVID-19), accurate resource allocation among hospitalized patients was necessary^3^.

While risk factors that predispose to serious consequences are now known^4^, the clinical deterioration of infected patients is still challenging to predict^5^. Therefore, early detection of patients at risk for rapid clinical deterioration is essential for patient management and better human and economic resource allocation^6^.

Machine learning (ML) enabled an effective prediction of outcomes that are not easily comprehended by other risk-predicting tools^7,8^. For example, XGBoost and RegCox algorithms have outperformed the widely used HAS-BLED clinical score in predicting the risk of bleeding^9^. Similarly, an ML model was developed to assess the risk of intubation among hospitalized patients with COVID-19 pneumonia^10^. In that study, model performance surpassed the previously validated ROX score, which incorporates oxygen saturation, respiratory rate, and the fraction of inspired oxygen to predict the risk of intubation^10^. Artificial neural networks (ANNs), a subtype of ML technique, have been used to stratify the risk of complex conditions such as syncope in the Emergency Department (ED) due to their ability to assess complex and non-linear relationships between predictors and clinical outcomes^11,12^. Additionally, ML-based models have been utilized to predict mortality or ICU admission related to COVID-19^13–15^.

Notably, most of those studies used imaging or laboratory data for outcomes prediction^16–20^, while only a few employed unstructured data obtained from electronic medical records (EMRs).

Natural language processing (NLP) is a useful technique in this context as it permits the analysis of medical charts written by nurses and clinicians^21^. This ML technology was already used during the pandemic for diagnosis^22^, radiological report analysis^21,23^, and patient features extraction^18,24^. Currently, NLP is mainly used for data mining, not outcome prediction^17^.

The current study hypothesized that integrating structured and unstructured data in an ML-based prediction model could provide valuable information on 30-day mortality and ICU admission for patients presenting to the ED with SARS-CoV-2 infection.

ANN algorithms combining textual and tabular data analysis of EMRs were developed to predict both outcomes in COVID-19 patients. Tabular models-based algorithms were used for comparison.

## Results

### Population characteristics

To train, validate, and test the ANNs we included 1296 SARS-CoV-2 patients who were admitted to the EDs of Humanitas Research Hospital (HRH, n = 509) and San Raffaele Hospital (SRH, n = 787). Of these, 252 died either during their hospital stay or within 30 days of discharge, and 158 were transferred to the ICU (Table 1).

**Table 1:** Demographic, clinical, radiological features and outcomes of COVID-19 patients by cohort.

	HRH and SRH cohort (n = 1296)	HRH cohort (n = 509)	SRH cohort (n = 787)
Demographic features			
Age, years, median (IQR)	63 (52–75)	67 (53–77)	61 (51–74)
Males, n (%)	825 (64)	322 (63)	503 (64)
Vital parameters			
Heart rate, bpm, median (IQR)	90 (80–103)	89 (79–100)	90 (80–104)
SAP, mm Hg, median (IQR)	130 (115–140)	130 (120–141)	126.5 (115–140)
DAP, mm Hg, median (IQR)	75 (65–80)	75 (65–80)	75 (65–80)
Oxygen saturation, median (IQR)	95 (90–97)	95 (90–97)	95 (92–98)
Respiratory rate, cycles per minute, median (IQR)	18 (16–24)	18 (17–20)	22 (16–30)
Temperature, °C, median (IQR)	37.1 (36.4–38)	37.0 (36.3–38.0)	37.2 (36.5–38.0)
Symptoms			
Duration from onset, days, median (IQR)	7 (4–10)	7 (2–12)	7 (4–10)
Fever, n (%)	1052 (81)	420 (83)	632 (80)
Cough, n (%)	672 (52)	290 (57)	382 (49)
Shortness of breath, n (%)	618 (48)	261 (51)	357 (45)
Diarrhea, n (%)	131 (10)	80 (16)	51 (6)
Fatigue, n (%)	114 (9)	39 (8)	75 (10)
Anosmia/Dysgeusia, n (%)	72 (6)	34 (7)	38 (5)
Nausea/Vomiting, n (%)	62 (5)	24 (5)	38 (5)
Myalgia/Arthralgia, n (%)	57 (4)	19 (4)	38 (5)
Asymptomatic, n (%)	48 (4)	34 (7)	14 (2)
Comorbidities			
Hypertension, n (%)	508 (40)	219 (43)	289 (37)
Diabetes, n (%)	219 (17)	101 (20)	118 (15)
Cardiovascular disease, n (%)	157 (12)	76 (15)	81 (10)
COPD, n (%)	72 (6)	38 (7)	34 (4)
Cerebrovascular disease, n (%)	79 (6)	38 (7)	41 (5)
Chronic kidney disease, n (%)	65 (5)	29 (6)	36 (5)
Malignancy, n (%)	72 (6)	28 (6)	44 (6)
Obesity, n (%)	62 (5)	24 (5)	38 (5)
Asthma, n (%)	48 (4)	13 (3)	35 (4)
No comorbidities, n (%)	323 (25)	171 (34)	152 (19)
Chest X-ray and CT scan			
Suggestive of COVID-19 pneumonia, n (%)	989 (76)	369 (75)	620 (79)
X-ray suggestive of COVID-19 pneumonia		48 (13.0)	614 (99.0)
CT scan suggestive of COVID-19 pneumonia		332 (89.9)	8 (1.3)
Outcomes			
30-day death, n (%)	252 (20)	119 (23)	133 (17)
ICU admission, n (%)	158 (12)	72 (14)	86 (11)

*HRH* stands for Humanitas Research Hospital; *SRH* San Raffaele Hospital; *bpm* beats per minute; *SAP* systolic arterial pressure; *DAP* diastolic arterial pressure; *COPD* chronic obstructive pulmonary disease; *CT* computerized tomography. There were missing data involving at least 5% of the studied population as far as respiratory rate (for both HRH and SRH), oxygen saturation (HRH) and temperature (SRH).

Table 1 displays the baseline characteristics of the two cohorts. The mean age was 63 years, with a male gender predominance. In both centers, the average duration of symptoms at the time of ED evaluation was 7 days. The most common symptoms were fever, cough, and shortness of breath. The most common comorbidities in both cohorts were hypertension, diabetes, cardiovascular disease, and COPD.

Radiological evidence of COVID-19 interstitial pneumonia was present in 76% of the population. The 30-day mortality rate was 20%, and the ICU admission rate was 12%. HRH had an older population and a higher mortality rate compared to SRH.

### Models performance

Table 2 summarizes the performance of fastai, TensorFlow and XGBoost models in predicting 30-day mortality in the test set. The TensorFlow tabular model showed an area under the curve (AUC) of 0.88 ± 0.02, an F1 score of 0.58 ± 0.04, and a Matthew's correlation coefficient (MCC) of 0.48 ± 0.05. The TensorFlow combined model performance had an AUC of 0.87 ± 0.06, an F1 score of 0.62 ± 0.10, and an MCC of 0.52 ± 0.13, which was slightly higher than the tabular model MCC (*p* = 0.36), indicating its prediction capability. The XGBoost and fastai models performed slightly worse than the TensorFlow models.

**Table 2:** Performance metrics of the models in predicting 30-day mortality and ICU admission.

	Specificity	Recall	Precision	NPV	F1-score	MCC	AUC
30-Day mortality							
TensorFlow tabular model	0.76 ± 0.03	0.76 ± 0.04	0.49 ± 0.03	0.93 ± 0.01	0.58 ± 0.04	0.48 ± 0.05	0.88 ± 0.02
Fastai tabular model	0.75 ± 0.06	0.77 ± 0.09	0.43 ± 0.04	0.93 ± 0.02	0.55 ± 0.03	0.43 ± 0.04	0.84 ± 0.02
XGBoost model	0.75 ± 0.06	0.77 ± 0.09	0.43 ± 0.04	0.93 ± 0.02	0.55 ± 0.03	0.43 ± 0.04	0.84 ± 0.02
TensorFlow tabular-textual model	0.82 ± 0.08	0.77 ± 0.13	0.53 ± 0.12	0.94 ± 0.03	0.62 ± 0.10	0.52 ± 0.13	0.87 ± 0.06
Fastai tabular-textual model	0.78 ± 0.07	0.74 ± 0.08	0.46 ± 0.06	0.93 ± 0.02	0.56 ± 0.04	0.44 ± 0.04	0.84 ± 0.02
ICU admission							
TensorFlow tabular model	0.72 ± 0.05	0.76 ± 0.10	0.27 ± 0.03	0.96 ± 0.01	0.40 ± 0.03	0.33 ± 0.05*	0.83 ± 0.04
Fastai tabular model	0.71 ± 0.06	0.70 ± 0.11	0.25 ± 0.03	0.95 ± 0.01	0.37 ± 0.04	0.29 ± 0.05	0.79 ± 0.04
XGBoost model	0.71 ± 0.06	0.70 ± 0.10	0.25 ± 0.03	0.95 ± 0.01	0.37 ± 0.04	0.29 ± 0.05	0.79 ± 0.04
TensorFlow tabular-textual model	0.92 ± 0.03	0.53 ± 0.15	0.48 ± 0.01	0.94 ± 0.02	0.49 ± 0.10	0.43 ± 0.11*	0.80 ± 0.07
Fastai tabular-textual model	0.72 ± 0.08	0.67 ± 0.11	0.25 ± 0.03	0.94 ± 0.01	0.36 ± 0.03	0.28 ± 0.05	0.79 ± 0.04

Results are presented as means and standard deviations of 10 iterations. *NPV*, negative predictive value; *MCC*, Matthew’s correlation coefficient; *AUC*, area under the curve. *p < 0.05 according to a paired *t*-test.

For ICU admission prediction, the combined TensorFlow model had highest specificity, F1, and precision scores. The TensorFlow combined model MCC was greater than the TensorFlow tabular model (*p* = 0.024) according to a paired *t*-test (Table 2). The TensorFlow tabular model performed better in terms of recall and AUC. The XGBoost model's performance metrics were slightly worse than those of the TensorFlow and fastai models.

### Models calibration

The calibration of each model was assessed using the expected calibration error (ECE). For the TensorFlow models, the mean ECE for predicting death outcome was 0.04 ± 0.006 for the tabular model, and 0.12 ± 0.04 for the combined tabular-textual model. For predicting ICU admission outcome, the mean ECE was 0.10 ± 0.43 for both tabular and combined models. The XGBoost model had an ECE of 0.10 ± 0.03 for 30-day mortality outcome and 0.06 ± 0.03 for ICU admission outcome. For the fastai models, the mean ECE was 0.10 ± 0.07 for the tabular model predicting death outcome and 0.10 ± 0.06 for the combined tabular-textual model. The mean ECE for predicting ICU admission was 0.06 ± 0.03 for the tabular model and 0.12 ± 0.05 for the combined tabular-textual model.

**Figure 1:**
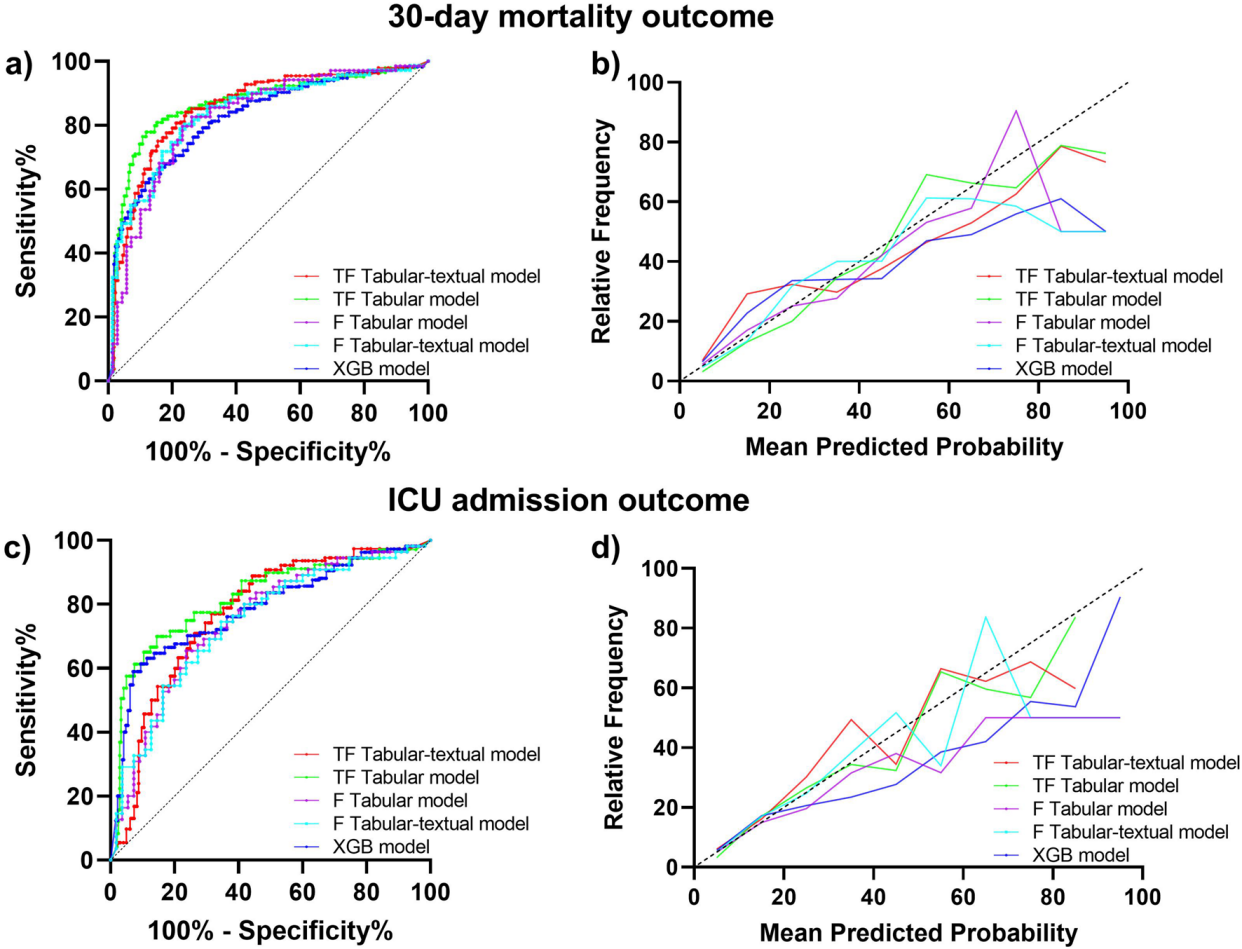
Models predictive performance and calibration. Fastai (F) tabular model performance is depicted as the purple line; fastai tabular-textual model is the cyan line; the XGBoost (XGB) model is the blue line; the TensorFlow (TF) tabular model is depicted as the green line; the TensorFlow model is the red line. (**a**) Receiver operating characteristic (ROC) curves of models for 30-day mortality outcome. (**c**) ROC curves of different models for ICU admission outcome. In both (**a**) and (**c**) the black dotted lines indicate the baseline, representing predictions of a random classifier. (**b**) and (**d**) depict the calibration curves of the models for 30-day mortality and ICU admission outcomes, respectively. For the calibration curves the black dotted line represents the model ideal calibration.

Figure 1 displays the receiver operating characteristic (ROC) curves and calibration of the models for predicting 30-day mortality and ICU admission.

## Discussion

The present study described the development of an algorithm that used textual and tabular data to predict 30-day mortality and ICU admission in patients in the ED for COVID-19.

The use of textual data for prognostic purposes was a novel aspect of this investigation.

The main finding was that combining tabular and textual variables led to better prediction of 30-day mortality compared to models using only tabular variables. The TensorFlow tabular-textual model had better performance metrics for 30-day mortality prediction, including specificity, precision, F1 score, and MCC, than the tabular models. For ICU admission, the combined model had higher precision, specificity, F1 score, and MCC values. Both models were adequately calibrated, with low ECE scores.

The methodology for analyzing textual data, i.e. the NLP, is commonly used for entity recognition, literature-based discovery, and question answering^25,26^. However, its potential for predicting outcomes in COVID-19 patients has not been fully explored. For example, Izquierdo et al.^24^, used NLP to identify COVID-19 patients from a general cohort and extract their clinical features. These were fed into a decision tree algorithm which identified ICU admission as the most relevant risk factor for adverse outcomes. Similarly, Graziani et al.^27^ explored the effects of COVID-19 on a population with COPD and used NLP to identify the population’s main features. Identified features were then correlated with the patient’s outcome by multivariable analysis and were associated with higher hospitalization and mortality rates. Ancochea et al.^28^ used NLP to identify patient features from EMRs and address the impact of gender on COVID-19 incidence and severity. The primary objective of the aforementioned investigations was to identify risk factors that could have influenced patient outcomes. In contrast to previous studies, the present study employed NLP analysis of textual data to process potential clinical information and predict 30-day mortality and ICU admission for COVID-19 patients. This highlights the importance of including textual data in COVID-19 prognosis prediction. Previous research by Silverman et al.^29^ also demonstrated the association between textual data and outcome prediction by using NLP techniques to extract COVID-19 symptoms and correlate them with in-hospital mortality and mechanical ventilation. These authors observed an association between textual data and outcome prediction, highlighting the importance of textual contribution. Similarly, the TensorFlow tabular-textual algorithm developed in this study was able to manage clinical information such as the patient's medical history, physical examination, specialist consultations, and radiological reports, which may provide crucial information related to clinical outcomes. In the past, manual annotations were required to optimize such an approach^30^, but the algorithm developed in this study more efficiently utilized the predictive power of textual variables contained in the EMRs without requiring manual revision.

According to the performance metrics presented in Table 2, our combined TensorFlow tabular and textual model was more effective than the models that used only tabular data in predicting 30-day mortality for COVID-19 patients. This finding is consistent with Sung et al.^31^ who compared the effectiveness of classic ML models with NLP-enhanced ML models in predicting acute ischemic stroke prognosis and found that incorporating textual predictors (i.e. history of present illness) improved the AUC metric of the algorithm. However, it is important to note that stroke is a well-studied disease, whereas COVID-19 was still a novel condition at the time of data collection. This could have led to healthcare professionals using more simplified language in their notes and radiology reports, resulting in a lower quantity of informative elements suitable for accurate outcome prediction.

The improved specificity observed in the current TensorFlow tabular and textual model, compared to simple tabular models, could have important clinical implications. For instance, it may help the ED physician in determining the optimal timing for initiating positive pressure ventilation^32,33^ and selecting the most suitable ward for the patient's disposition, such as general medicine, sub-intensive, or ICU, based on the predicted prognosis. Additionally, the greater precision values of the combined algorithm, resulting in fewer false positives, may help distinguish between patients who require hospital admission and those who are eligible for discharge from the ED, contributing to resource allocation optimization. The F1 score and MCC values, which provide a more comprehensive evaluation of the algorithm's prediction, further support these findings. Therefore, integrating textual and tabular data may improve the prediction of negative outcomes in COVID-19 patients.

ML algorithms typically employ tabular data to predict outcomes in various diseases, including sepsis and upper gastrointestinal bleeding^26,27^. Since the emergence of SARS-CoV-2, numerous tabular data-based ML models have been developed for COVID-19 early recognition, diagnosis, including interstitial pneumonia from chest radiography (X-ray) images, and prognosis^28,29,31,34–36^.

The mean performances of our TensorFlow tabular and XGBoost models were found to be consistent with previous literature findings^16,37–39^. The AUC value of the current TensorFlow tabular model was higher than that of Vaid et al., who used a cohort from the initial outbreak in New York City. However, that study focused on predicting COVID-19 10-day mortality^32^. In comparison to the current study findings, Gao et al.^33^ obtained greater AUC, sensitivity, specificity, and an F1 score using a neural network-based algorithm similar to ours, but they used more predictors, which may have led to the overfitting of their model^40^.

The selection of predictors may account for the differences in the prognostic prediction ability of various tabular models. In our study, age, plasma creatinine, C-reactive protein, hemoglobin, and platelet values were used to develop the TensorFlow tabular model. They were chosen based on previous research findings^41–48^ and their prompt availability in our emergency settings. However, other variables such as myoglobin, ferritin, and troponins^47,49,50^ have also been found to affect COVID-19 prognosis.

Our data were obtained from the EDs of two academic hospitals, which provided a larger cohort for model training and validation and improved the algorithm's applicability to other cohorts. However, we had to overcome the challenge of differences in the structure of EMRs and radiological reports, as well as slight variations in language use between the two centers. The latter involves variations in the use of single terms and language syntax.

Regarding ICU admission prediction, our results indicate that the TensorFlow tabular-textual model outperforms those that use simple tabular variables. It is important to highlight the complexity of predicting ICU admission, as there are confounding factors that are not necessarily related to disease severity, such as lack of available beds and mechanical ventilators, which occurred frequently during the early phase of the first COVID-19 outbreak in Italy^36^. Other studies have considered ICU admission as an adverse outcome after COVID-19 pneumonia. For example, Li et al.^51^ developed a convolutional neural network with ICU admission being the primary COVID-19 outcome. They showed similar but not identical performances compared to the models developed in the present study. Differences from our results can be accounted for by the larger proportion of patients admitted to the ICU and the different predictors used in their study.

Finally, the low ECE scores for both models suggest that our algorithms perform with high confidence, which is crucial for ED physicians in making safe decisions on whether to admit or discharge patients.

### Limitations and future perspectives

A limitation of the present study is that external validation is needed to confirm the reliability of the results beyond the two centers where the data was collected. Moreover, the interpretability of models is limited due to the intrinsic nature of neural networks, making it impossible to establish a causal relationship between predictors and outcomes.

Furthermore, since the current data set was collected during the early phase of the pandemic in northern Italy, outcomes prediction is confined to the specific features of the circulating virus and therapies available at that time. Additionally, the absence of vaccinated patients in the dataset means that the potential protective effect of vaccines is not reflected in the results.

From a clinical perspective, it is believed that applying the same algorithms to data collected during different waves of COVID-19 could provide valuable information on vaccine efficacy and the evolution of the disease with new variants. In the future, combining textual and tabular data analysis could help to clarify the clinical progression of COVID-19 in hospitalized patients with new variants, potentially optimizing antiviral therapies.

Finally, the algorithms combining textual and tabular predictors are not limited to COVID-19 prognosis prediction. If supplied with new data, the language model can be trained to analyze EMRs related to other disorders commonly encountered in the ED, such as heart failure, sepsis, pneumonia, COPD, syncope, and others. When dealing with these diseases, emergency physicians could better stratify patient risk and make more informed decisions regarding admission or discharge. Ultimately, this could lead to better utilization of hospital resources and healthcare system efficiency.

### Conclusions

The results of this study suggest that the combined analysis of tabular and textual data was effective in predicting 30-day mortality in patients with SARS-CoV-2 infection presenting to the ED and in predicting their ICU admission. The combination of tabular and textual data represents a novelty, at least partially^24^, in the clinical research dealing with the COVID-19 outbreak.

Our findings suggest that NLP may also have a potential application for prognosis prediction in other common diseases seen in the emergency setting, as noted in a recent systematic review^26^.

Future studies will be necessary to improve the accuracy of the algorithm and confirm its generalizability through external validation cohorts, especially with the changing epidemiology of the pandemic.

## Methods

### Study population and outcomes

In this bi-centric retrospective cohort study, EMRs of all confirmed COVID-19 cases evaluated at the ED of HRH and SRH, in the Milan area, between February 20 and May 5, 2020, were analyzed.

Confirmed COVID-19 cases were defined as patients who tested positive for the SARS-CoV-2 virus by real-time reverse-transcription PCR assay on a nasal and pharyngeal swab, broncho-alveolar lavage, or broncho-aspiration specimens performed in the ED. Patients admitted to ICU on arrival at the ED were excluded.

Data collected included: triage information, diagnosis, medical history, physical examination, clinical notes, vital parameters, blood tests, imaging exam reports, and ED disposition. Both tabular (e.g. vital parameters, blood exams) and free-text (e.g. clinical notes and radiological reports) data were used.

The primary COVID-19-related outcome was the 30-day mortality rate, and the secondary outcome was ICU admission.

### Model development

EMRs of 15,599 patients evaluated in the two EDs over the study period were considered to develop the ANN (i.e. fastai, TensorFlow) and decision tree (i.e. XGBoost) models.

**Figure 2:**
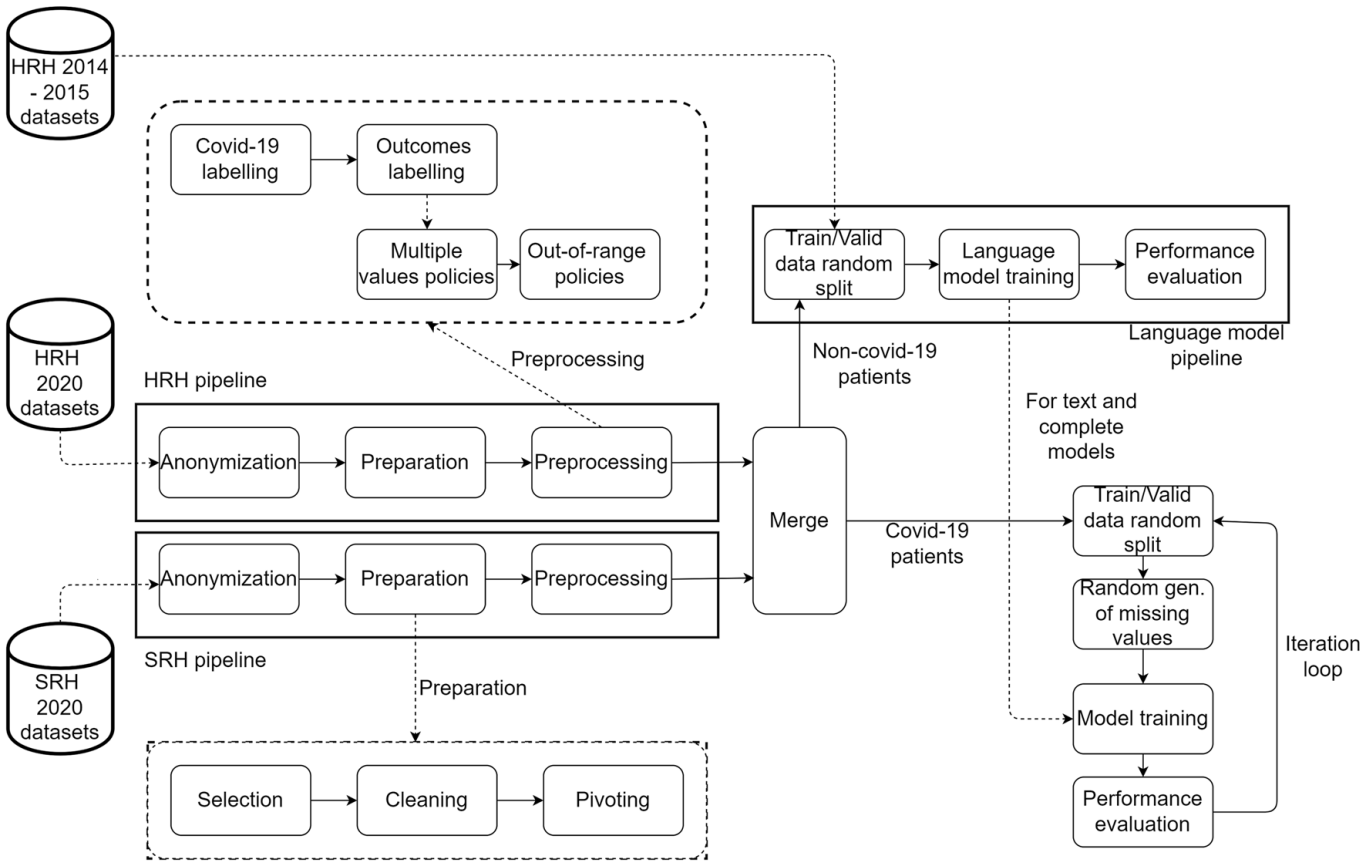
Algorithm pipeline for model development. Cylinders represent datasets. Boxes are algorithms or groups of algorithms. Solid arrows indicate the data/computation flow. Dashed arrows show the internal organization of groups of algorithms. Data collected from Humanitas Research Hospital (HRH) and San Raffaele Hospital (SRH) are processed by two separate groups of algorithms and merged into a single dataset. These two groups of algorithms performed data anonymization, preparation, and preprocessing. Once the datasets were merged, patients without COVID-19, jointly from the data of a previous study^30^, were selected to train, validate, and test the language model. Likewise, patients with COVID-19 were used for the predictive models. The preparation group algorithms select the clinically relevant predictors (selection), clean the data (cleaning) and transform the data so that for each patient, all the data are in a single row (pivoting). The preprocessing group algorithms identify the patient with COVID-19, those that died, and those that were transferred to ICU (labeling). Next, the sequence of values is converted into a single value by multiple values policies, and the out-of-range policies handle values that exceed the expected range of values.

Data from each hospital was first processed independently, as shown in Fig. 2.

In both algorithm pipelines, the first step was anonymization (e.g. secure hashing of patient identifiers); the second step was preparation, and the third was preprocessing.

The preparation phase included three sub-steps: features selection, data cleaning, and pivoting. Features selection was based on potential risk factors previously identified in scientific literature. In the pivoting step, the dataset was organized such that all data for a patient were in a single row and each variable was in a single column.

**Figure 3:**
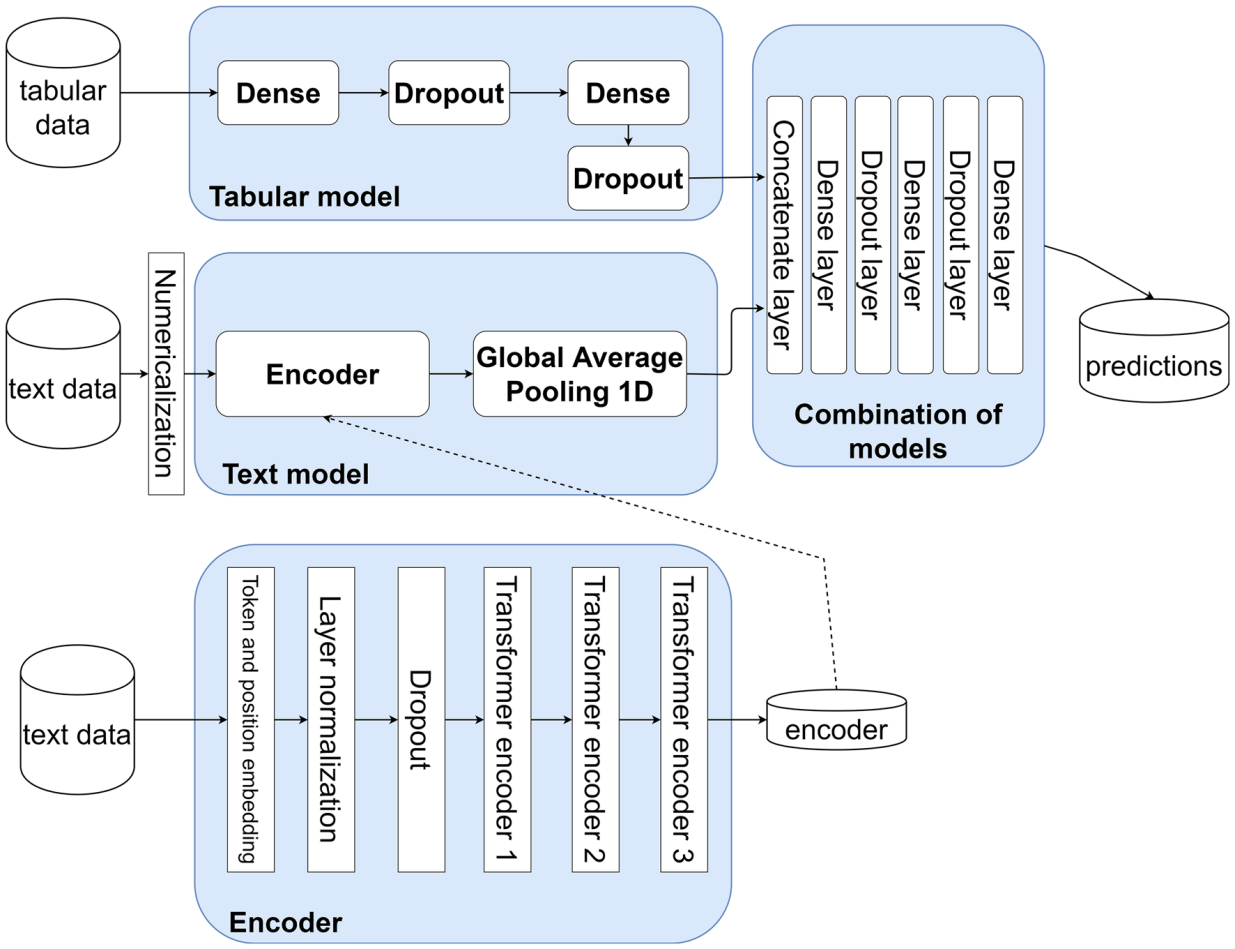
TensorFlow tabular-textual model architecture. Cylinders represent datasets, and boxes are algorithms or groups of algorithms. Solid arrows indicate the data/computation flow. The tabular data of each study subject go through the tabular model, while the text data are first ‘numericalized’ and then are sent to the textual model. The tabular and textual models are taken from the fastai 1 library: hyperparameters are tuned, but the architecture is unchanged. The tabular and textual model outputs go through a network consisting of nine layers (a combination of models) of four different types: linear layers, ReLU (Rectified Linear Unit) activations, batch normalization, and dropout layers. The number of layers, their type, and the number of neurons in the combination of models section are kept identical in all experiments.

The preprocessing phase consisted of four main steps, as depicted in Fig. 2. Firstly, COVID-19-positive patients were singled out of all those in the initial dataset (i.e. all patients admitted to the two EDs during the study period). Then, patients were categorized based on outcomes of death and ICU transfer. After that, outlier policies were applied, where outliers were replaced with boundary values (e.g. heart rate values above 250 bpm or below 20 bpm were replaced with a default boundary value). Multiple values policies were employed, converting each patient’s data sequence into a single number by taking the first valid (not null) data item. Lastly, out-of-range values were handled according to specific rules as required (e.g., C-reactive protein values below the normal range were reported as “< 0.08”: these values were substituted with the lower limit value). After being processed separately, the datasets from both hospitals were merged into a single dataset. The merged dataset was subsequently used to train the ANN and XGBoost models, as shown in Fig. 3.

The textual data of all COVID-19-negative patients in the merged dataset was joined to the textual data of a previous study dataset^30^. This purely textual dataset was used to train the language model. A statistical language model is a probability distribution over a sequence of words. It can be estimated, for instance, by fitting an ANN and can be used to get a numerical representation of the text while preserving the semantic relations.

The language model was then used to improve the performance of COVID-19 prognosis prediction models whenever textual data was considered. However, none of the textual data of COVID-19-positive patient data was used to train or validate the language model.

The COVID-19-positive patients’ data in the merged dataset was subsequently randomly split into train, validation, and test sets. Ten different random splits were performed, and for each, the model training was carried out, and the performance was evaluated. The data splitting, model training, and model evaluation cycle is schematized in Fig. 2 as ‘iteration’.

For each split and metric, the mean and standard deviation of the values obtained by the iteration was provided. All models were used to predict the likelihood of the target outcome (e.g. death and ICU admission). A 0.50 threshold was applied to convert the likelihood into a binary prediction (e.g. a likelihood of 0.51 means a prediction of having outcome 1). No other thresholds were used.

Notably, in each iteration, three distinct models were trained and evaluated as illustrated in Fig. 1: (i) a tabular model, whose input data was only the selected numeric predictors (i.e. age, creatinine, C-reactive protein, hemoglobin, and platelets); (ii) a text model, whose input data was only text predictors (i.e. history, physical exam, and radiological reports); (iii) a tabular-textual model, whose input data was both numeric and text predictors.

In each iteration, the fastai models were trained on the training dataset by gradually unfreezing the model layer parameters^52^. Gradual unfreezing is used to reap the benefits of a transfer learning design pattern^53^. This pattern is based on the idea of applying to a specialized task (i.e. COVID-19 prognosis) for which there is little data, the weights of a model trained for a different task (i.e. the Italian medical language model) for which more data is available.

Training of the fastai tabular model was performed by freezing all but the last layer and fitting for two epochs. Then, all but the last two layers were frozen and fitting was performed for two more epochs. Finally, all layers were unfrozen, and fitting was executed for ten epochs. The best model (i.e. the model with the highest value of the reference performance metrics on the validation set, see below) obtained during training was selected and used to compute the performance on the test set. A similar approach was used to train the tabular-textual model but with a slightly different number of epochs and different layers frozen.

All fastai models were trained using Cross Entropy Flat Loss function^54^ and AdamW optimizer^55^. In each iteration, missing tabular training data values were randomly generated using a normal distribution based on the training data values, but only when the percentage of missing values did not exceed 5%.

TensorFlow models were all trained with the CrossEntropy loss function and the AdamW optimizer. A detailed description of the tabular-textual model architecture and of learning hyperparameters is contained in the supplementary appendix.

Models were built using the fastai v. 1^56^, the TensorFlow and the XGBoost frameworks.

Language model training on a MacBook Pro (processor 2.3 GHz 8-Core Intel Core i9, RAM 16 GB) required 144 h to achieve a perplexity of 11.28 and an accuracy of 51.89% on the language model validation set. The 144-h computational time refers to the training time of the language model used in the textual and text-tabular models. The model inference time, lasting less than one second, is the time that may matter to the physician in the ED. This means that the computation time can be used profitably by emergency doctors.

Such results were achieved without hyperparameter tuning using the fastai v.1 language model.

### Performance measures

To evaluate the predictive performance of each model, discrimination and calibration were assessed. In addition to the F1 score, C-index, and AUC calculation, MCC was chosen as a reference metric to identify the best model on the validation set due to its intrinsic ability of simultaneously considering true positive, false positive, true negative, and false negative predictions, making it more reliable for binary classification tasks^57^. MCC ranges from − 1 to + 1, with 0 representing a prediction no better than random. A paired *t*-test was used to compare the MCC computed for each model on test sets.

Model calibration addresses the likelihood of correct predictions represented by the predicted probability estimates. This metric discretizes the probability interval into a fixed number of bins and assigns each predicted probability to the bin that encompasses it. ECE is the difference between the fraction of correct predictions in the bin (accuracy) and the mean of the probabilities in the bin (confidence). Therefore, if a model’s accuracy is equal to the bin’s mean probability, the ECE would be 0, indicating perfect calibration. Lower values of ECE correspond to better calibrations^58,59^. When a scalar summary statistic is insufficient, calibration is often visualized using reliability diagrams plotting the expected sample accuracy as a function of confidence.

Training the models for all ten necessary iterations required 47 sec for the tabular model, 18.38 h for the text model, and 82 h for the combined model. The training was performed using fixed learning rates for the fastai tabular model and discriminative learning rates for the other fastai models^56,60^. For TensorFlow models see the supplementary appendix.

All methods were conducted in accordance with the Helsinki declaration. All experimental protocols were approved by the Institutional Review Board of Humanitas Clinical and Research Center—IRCCS, IRB approval number 2544; May 20, 2020. Informed consent was obtained from all subjects or their legal guardian(s).

## Supplementary Information

Supplementary Information.

Supplementary Legends.

Supplementary Tables.

## Data Availability

Publication of data would compromise individual privacy. Please contact RF for disclosure.
