# Supplementary material for: Multimodal deep learning for COVID-19 prognosis prediction in the emergency department: a bi-centric study
Source: Sci Rep. 2023 Jul 5;13:10868. doi: 10.1038/s41598-023-37512-3 (PMC10322913; doi:10.1038/s41598-023-37512-3)
Supplement: Supplementary file 1 — Supplementary Information. [file 41598_2023_37512_MOESM1_ESM.docx]

**Multimodal deep learning for COVID-19 prognosis prediction in the emergency department: a bi-centric study.**

*Variable Selection*

Predictors for the tabular model were selected through a three-step process. First, literature review was performed to determine a list of the most important predictors for mortality and ICU admission. Then, we cross-checked the list with available data in our dataset and identified possible candidates for the tabular model predictors. Finally, variables with the least missing values were selected being: age, C-reactive protein, platelets, creatinine and hemoglobin.

Textual variables obtained from EMRs were patient history, physical exam and radiological reports of imaging performed in the ED.
