# Supplementary material for: Multimodal deep learning for COVID-19 prognosis prediction in the emergency department: a bi-centric study
Source: Sci Rep. 2023 Jul 5;13:10868. doi: 10.1038/s41598-023-37512-3 (PMC10322913; doi:10.1038/s41598-023-37512-3)
Supplement: Supplementary file 2 — Supplementary Legends. [file 41598_2023_37512_MOESM2_ESM.docx]

Supplementary table 1. Architecture of tabular-text model used for outcome ‘death’

For the tabular-textual model predicting 30-day mortality, total parameters were 2,923,649. The trainable parameters were 2,923,649. There were no non-trainable parameters. Hyper-parameter training was performed with the AdamW optimizer. The learning rate used in all layers was 0.001.

Supplementary table 2. Architecture of tabular-text model used for outcome ‘ICU’

For the tabular-textual model predicting ICU admission, total parameters were 2,900,993. Trainable parameters were 2,900,993. There were no non-trainable parameters. Hyper-parameter training was performed with the AdamW optimizer. The learning rate used in all layers was 0.0001 except for three layers. The first_tab_text_dense_layer, second_tab_text_dense_layer and the encoder_model layer had a learning rate of 0.001. A default value was used as Weight decay in all layers except for two layers. In the first_tab_text_dense layer and second_tab_text_dense_layer Weight decay was 0.01.
