# Supplementary material for: Multimodal deep learning for COVID-19 prognosis prediction in the emergency department: a bi-centric study
Source: Sci Rep. 2023 Jul 5;13:10868. doi: 10.1038/s41598-023-37512-3 (PMC10322913; doi:10.1038/s41598-023-37512-3)
Supplement: Supplementary file 3 — Supplementary Tables. [file 41598_2023_37512_MOESM3_ESM.docx]

# **Supplementary table 1. Architecture of tabular-text model used for outcome ‘death’**

| Layer name | Layer type | Output shape | Parameters |
| --- | --- | --- | --- |
| input_tab_layer | InputLayer | [(None, 5)] |  |
| first_tab_hidden_layer | Dense | (None, 128) | 768 |
| first_tab_dropout_layer | Dropout | (None, 128) | 0 |
| second_tab_hidden_layer | Dense | (None, 128) | 16512 |
| second_tab_dropout_layer | Dropout | (None, 128) | 0 |
| input_text_layer | InputLayer | [(None, 165)] | 0 |
| global_averaging_pool_text_layer | GlobalAveragePooling1D | (None, 256) |  |
| tab_text_concatenation_layer | Concatenate | (None, 384 | 0 |
| first_tab_text_dense_layer | Dense | (None, 128) | 49280 |
| first_tab_text_activation_layer | Activation | (None, 128) | 0 |
| first_tab_text_dropout_layer | Dropout | (None, 128) | 0 |
| outputs_tab_hidden_layer | Dense | (None, 1) | 129 |

# **Supplementary table 2. Architecture of tabular-text model used for outcome ‘ICU’**

| Layer name | Layer type | Output shape | Parameters |
| --- | --- | --- | --- |
| input_tab_layer | InputLayer | [(None, 5)] |  |
| first_tab_hidden_layer | Dense | (None, 128) | 768 |
| first_tab_dropout_layer | Dropout | (None, 128) | 0 |
| second_tab_hidden_layer | Dense | (None, 128) | 16512 |
| second_tab_dropout_layer | Dropout | (None, 128) | 0 |
| input_text_layer | InputLayer | [(None, 165)] | 0 |
| global_averaging_pool_text_layer | GlobalAveragePooling1D | (None, 256) |  |
| tab_text_concatenation_layer | Concatenate | (None, 384 | 0 |
| first_tab_text_dense_layer | Dense | (None, 64) | 24640 |
| first_tab_text_activation_layer | Activation | (None, 64) | 0 |
| first_tab_text_dropout_layer | Dropout | (None, 64) | 0 |
| second_tab_text_dense_layer | Dense | (None, 32) | 2080 |
| second_tab_text_activation_layer | Activation | (None, 32) | 0 |
| second_tab_text_dropout_layer | Dropout | (None, 32) | 0 |
| outputs_tab_hidden_layer | Dense | (None, 1) | 33 |
